# Encapsulation of *Lactobacillus rhamnosus* GG in edible electrospun mats from calcium and sodium caseinates with pullulan blends

**DOI:** 10.3168/jdsc.2021-0173

**Published:** 2022-10-21

**Authors:** Serife Akkurt, John Renye, Peggy M. Tomasula

**Affiliations:** Dairy and Functional Foods Research Unit, Eastern Regional Research Center, Agricultural Research Service, USDA, Wyndmoor, PA 19038

## Abstract

Electrospinning has been proposed as a method to encapsulate and preserve bioactive compounds in nanofibrous mats to ensure their delivery and associated health benefits when consumed directly or added to a food formulation. In previous work, we demonstrated the production of edible fibers to form mats of both calcium (CaCAS) and sodium (NaCAS) caseinate-pullulan (PUL), with the polysaccharide PUL added as a carrier to facilitate molecular entanglement for fiber formation. In this study, we determined the viability of the probiotic bacteria, *Lactobacillus rhamnosus* GG (LGG), used as a model bacterium, in mats of CaCAS-PUL and NaCAS-PUL. Electrospinning of aqueous solutions at room temperature (21 ± 1°C) of 15% (wt/wt) CaCAS and NaCAS mixed with 15% (wt/wt) PUL, with a 1:1 ratio of CAS:PUL, resulted in fibrous mats with average fiber diameter sizes of 233 ± 20 and 244 ± 21 nm, respectively, as determined by scanning electron microscopy. Addition of LGG in the amounts of 9.3 and 9.0 log_10_ cfu/mL to the CaCAS-PUL and NaCAS-PUL solutions before electrospinning resulted in average fiber diameter sizes of 212 ± 14 and 286 ± 16 nm, respectively. The LGG was found to be distributed within the CaCAS-PUL and NaCAS-PUL fibers. The addition of LGG increased the shear viscosity and conductivity of the CaCAS-PUL solution, enhancing molecular entanglement and resulting in thinner fibers. For NaCAS, LGG increased the conductivity but reduced shear viscosity. Adjustment of the NaCAS-PUL composition would be needed to optimize conditions for thinner fibers. The numbers of viable LGG recovered from the CaCAS-PUL and NaCAS-PUL nanofibrous mats after electrospinning were 9.5 and 9.6 log_10_ cfu/g, respectively, proving that the electrospinning conditions used were capable of supporting probiotic encapsulation. These results demonstrate that food-grade electrospun fibrous mats can be used to develop functional foods with delivery of probiotics to improve human or animal health.

## Highlights

• Electrospun fibers were prepared from Ca or Na caseinates with pullulan in solution.
• The 3-dimensional mats were made at room temperature conditions that included spaces within and outside of the fibers.
• Including LGG in the solutions caused attachment of the bacteria to the fibers.
• High viability of the LGG was shown after electrospinning.

## Graphical Abstract

**Figure:**
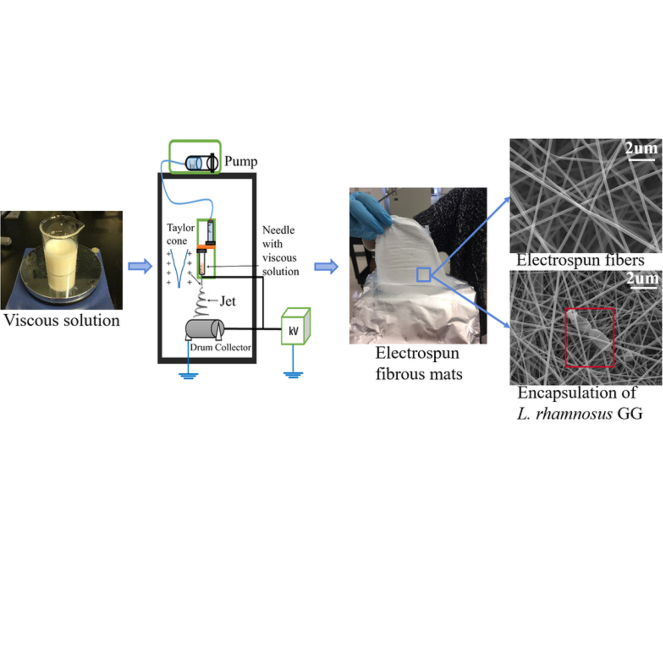
**Summary:** Electrospinning is a process that applies an external voltage to a viscous solution to produce 3-dimensional fibrous mats, making them an ideal format for preservation of bioactives such as probiotics. The viability of probiotics can lessen during processing and storage. In this study, the probiotic bacteria *Lactobacillus rhamnosus* GG (LGG) was electrospun with a caseinate-based solution and encapsulated within a mat. The LGG was enveloped by the fibers as they were produced, which protected its viability. Preservation of LGG in this manner could allow for development of functional foods that enhance the delivery of probiotics.

Probiotics are defined as “live micro-organisms, which when administered in adequate amounts, confer a health benefit on the host” (FAO/WHO, 2002), with amounts in the ranges of 10^6^ to 10^8^ cfu·g^−1^ or 10^8^ to 10^10^ cfu/d suggested to promote health benefits (Champagne et al., 2011). The genus *Lactobacillus* includes many species that are used as probiotics in dairy products such as yogurt, kefir, ice cream, and milk, and nondairy products, such as fruit juices, chocolate, and fiber snacks (Anal and Singh, 2007; Saad et al., 2013; Burgain et al., 2015; Terpou et al., 2019). However, maintaining the viability of probiotics during production and product shelf life is challenging due to intrinsic (species and strain types) and extrinsic factors (low pH, oxygen, water content, or water activity, processing conditions) (Espitia et al., 2016; Terpou et al., 2019). Several techniques have been proposed to maintain viability of probiotics. These are mainly dehydration techniques (spray or freeze-dried systems) and incorporation of probiotics in microcapsules or in edible films were demonstrated for the production of viable probiotics in real foods or food packaging (Soukoulis et al., 2014; Espitia et al., 2016). However, dehydration techniques cause excessive stress on the probiotic cell membrane, reducing the viability of probiotics. The increasing expectations of consumers for healthy and functional food products drive research to create new systems or methods to overcome these problems (Burgain et al., 2015).

Electrospinning is a nonthermal technology produces nonwoven mats from nanoscale fibers by applying an electric field to a polymer solution. Electrospun mats have been extensively used in a wide range of applications due to their unique properties including a higher porosity, large surface area per unit mass or volume, higher permeability, and small intrafibrous pore size (Greiner and Wendorff, 2007). These properties provide a new perspective on the delivery of bioactive compounds and development of composite materials for filtration and active food packaging (Drosou et al., 2017). In addition to these applications, one of the potential uses for edible electrospun nanofibrous mats is to design functional foods by using the mats as a delivery vehicle for sensitive bioactives such as probiotics.

The electrospinning process is simple and consists of 4 main components, which are a pump, a syringe to contain a polymer solution linked to a needle, an external voltage supply, and a grounded collector or counter electrode for collecting the fibers in a fibrous mat. The solution forms a pendant drop at the needle tip of the syringe. The drop is balanced at the tip by the surface tension of the solution and is ejected when an electrostatic force overcomes the surface tension (Shenoy et al., 2005; Greiner and Wendorff, 2007). The applied electric field stretches the pendant drop into a cone shape, called a Taylor cone, which then forms a continuous jet. As the jet moves toward the grounded collector, it continues in an unstable whipping motion stretching and becoming thinner as the solvent evaporates, leading to randomly oriented fibers in the form of a fibrous mat on the grounded collector (Fong et al., 1999).

The electrospinning of synthetic and water-soluble polymers dissolved in organic solvents has been extensively studied and reviewed in the literature for biomedical and other applications (Huang et al., 2003; Tan et al., 2005; Wendorff et al., 2012). There are a few examples of food-based polymers including polysaccharides and proteins electrospun from their aqueous solutions for food use. Proteins dissolved in water are challenging to electrospin because of their 3-dimensional structures including complex secondary (i.e., α-helix, β-sheets), tertiary structure, and globular structure, which have less and weak interactions with each other to stretch and entangle under an electric field (Nieuwland et al., 2013; Akkurt et al., 2018).

A wide variety of casein-based food and pharmaceutical delivery systems utilizing the properties and functions of casein have been studied in the forms of edible films (Bonnaillie et al., 2014), hydrogels (de Kruif et al., 2015), emulsions (Giroux, Robitaille, Britten, 2016), and micro and nanoparticles (Semo et al., 2007; Jarunglumlert et al., 2015). Tomasula et al. (2016) reported another delivery method that was edible electrospun fibrous mats/fibers, successfully produced from aqueous calcium caseinate (**CaCAS**) and sodium caseinate (**NaCAS**) with pullulan (**PUL**), a food-grade polysaccharide as a carrier to facilitate formation of the caseinate fibers. Pullulan is a linear and noncharged food-grade polysaccharide with repeating units of both maltotrioses linked by α-(1→6) linkages and glucopyranose connected by α-(1→4) glucosidic bonds (Kong and Ziegler, 2014; Tomasula et al., 2016). Mitsuhashi et al. (1990) reported that PUL could have potential prebiotic properties for enhancing the activity of some probiotic strains in the colon due to the production of PUL-degrading enzymes by *Lactobacillus* and *Bifidobacterium*. Also, studies have used PUL as a prebiotic in food formulations such as yogurt to strengthen the growth of probiotics and extend their viability (Chlebowska-Śmigiel et al., 2019; Kycia et al., 2020). However, limited studies have focused on the possibility of using the combination of CAS and PUL to produce electrospun nanofibers to encapsulate LGG and prolong its viability. Due to the electrostatic, hydrophobic, and hydrophilic nature of caseinate proteins, the electrospun fibrous mats produced from caseinate (**CAS**)-based solutions have a high potential to increase the stability of *Lactobacillus rhamnosus* GG (**LGG**) and provide hydrophobic pockets that protect the bioactive cells from adverse surrounding conditions (Burgain et al., 2013a). The objective of this study was to evaluate the incorporation of LGG within fibers of the fibrous mats produced from aqueous CaCAS blended with PUL solutions to investigate the viability of the loaded probiotics.

The CaCAS and NaCAS were purchased from the American Casein Co. (AMCO Proteins). The CAS contains 88% as measured, or 90% protein (dry basis) as provided by the manufacturer. The PUL was purchased from TCI America. De-ionized (**DI**) water produced by a Milli-Q Synthesis water purification system (Millipore) was used to prepare all solutions. Aqueous solutions of 7.5% and 15% (wt/wt) CaCAS or NaCAS, and PUL were prepared at room temperature (**RT**; 21 ± 1°C). Then, 15% (wt/wt) CAS solutions were mixed with 15% (wt/wt) PUL solutions (pH ∼6.7), with a 1:1 weight ratio for 2 h at RT under continuous mixing with a magnetic stirrer (Cole-Parmer) to make blend solutions with a total concentration of 15%. The solutions were refrigerated overnight at 4°C to remove air bubbles. Before electrospinning, they were removed from the refrigerator and slowly mixed for about 20 min to reach RT before electrospinning.

The shear viscosities of the solutions as a function of shear rate from 0.1 to 1,000 s^−1^ at 20°C were measured using a rheometer (Kinexis Lab+; Kinexus Instruments) equipped with a 27.4 mm inside diameter cup and a 25 mm outside diameter bob. The shear viscosity at 100 s^−1^ was used as an indicator to predict that fiber production is possible for the solution system (Kong and Ziegler, 2014).

The electrical conductivities of the solutions were measured using a conductivity meter (IQ270G; Scientific Instruments) with a built-in temperature sensor and automatic temperature correction (reference temperature = 25°C). All measurements were conducted at RT. For PUL solutions, the conductivity results were close to the detection limit of the device (0.01 mS/cm), and thus the test required repeats to indicate a range of values (n = 10).

The surface tensions of the solutions were determined (n = 10) using a ramé-hart Advanced Automated Goniometer equipped with automated tilt, dispensing, and DROPimage advanced (model 590 F4, ramé-hart Instruments).

The zeta potentials (**ZP**) of the solutions, diluted from 15% to 5% (wt/wt) with DI water, were determined by the Zetasizer Nano series (Nano Z, Malvern Instrument Inc.) at an applied voltage of 150 V. Because DI water was used as a solvent in this study, it was chosen as a diluent for the ZP analysis, and the samples were diluted from 15% or 5% (wt/wt) to measure the ZP. The samples were measured in a folded capillary cell with a capacity of 1.2 µm solution (n = 3). The ZP was determined from the electrophoretic mobility based on Smoluchowski's formula (Huppertz and Fox, 2006). The ZP analysis as a function of pH from ∼7.0 to 10.0 adjusted by 1 *M* NaOH was performed on the solutions at RT (21°C).

The electrospinning process was conducted as described previously (Tomasula et al., 2016), with some modifications. Each CaCAS or NaCAS-PUL solution was fed into a 3-mL disposable plastic syringe fitted with a tubeless spinneret in an electrospinning unit with a syringe pump, a high voltage generator, and a grounded rotating cylinder receptor (model TL-Pro, NaBond Technologies). The pump delivered the solution to the needle at a flow rate of 3 mL/h. The voltage source, with a range of 0 to 50 kV, was connected to the needle, and the fiber was collected on the rotating cylinder (10 cm diameter) covered with Reynolds Wrap Non-Stick aluminum foil. All samples were electrospun at RT under constant operating parameters: the distance from the needle tip to the receptor was 12 cm; voltages were adjusted between 15 to 20 kV; the rotation speed of the collector was 80 to 84 revolutions/s; and the x-axial sliding speed of the needle was 10 to 13 mm/s. The dimensions of the rotating cylinder were 100 × 250 mm (diameter × length). The fibrous mats were obtained by moving the needle side to side over a distance of 10 cm. The fibrous mats with average dimensions of 20, 10, and 0.01 cm (width, length, and thickness, respectively) were produced by using 3 mL of each solution.

The mats produced without LGG were used for scanning electron microscopy analysis in 2 d. They were stored in a desiccator cabinet (30.5 × 61 × 30.5 cm, Plas-Labs Inc.) with a desiccant (≥98% CaSO_4_, Drierite, W.A. Hammond Drierite Co.) at RT to keep them from moisture.

Each mat was peeled from the foil, and a small strip (2 × 2 mm) was removed and coated with a thin gold film to produce a high-quality image for scanning electron microscopy analysis (model Quanta 200 F, FEI; Tomasula et al., 2016). The high-vacuum secondary electron imaging modes with a distance of 12.5 mm were employed at an accelerating voltage of 10 kV. ImageJ software with the DiameterJ plugin (ImageJ) was used to construct a histogram of the fiber diameters and to determine the pore size or space among the fibers.

*Lactobacillus rhamnosus* GG (ATCC 53103) was maintained in de Man, Rogosa, and Sharpe (**MRS**) broth (Difco Laboratories) at 37°C. Before inoculation into the 3 mL of 15% (wt/wt) CaCAS-PUL or NaCAS-PUL solutions, LGG was grown overnight in 25 mL of MRS broth, which reached a cell density of 4 × 10^9^ cfu/mL. Cells were collected by centrifuging at 2,990 × *g* for 10 min at RT, washed 3 times in 0.1% peptone water, and then resuspended in 5 mL of each CAS-PUL solution (pH ∼7.0). The LGG viability was assessed by plate count on MRS agar immediately after resuspension in the CAS-PUL solutions to determine the initial microbial load in each CAS solution in cfu/mL.

Electrospinning was carried out as described above. Mats produced with LGG were immediately assessed for cell viability. To determine the viability of LGG within the nanofibrous mats, 1 g of the mats was dissolved in 9 mL of peptone water (0.1%) by shaking at 2,200 rpm for 1 h at RT, and then colony counts were performed on MRS agar following incubation for 2 d at 37°C. The LGG cells within the mats were visualized using scanning electron microscopy.

Statistical analysis of the data was performed using the OriginLab software package with its statistical analysis plugin (OriginLab Corporation), and the results are presented as means ± standard deviation. One-way ANOVA and Tukey's test (*P* < 0.05) were used to determine the significant differences between fiber diameter sizes.

Scanning electron microscopy images for electrospinning of the 7.5% (wt/wt) aqueous solutions of CaCAS, NaCAS, and PUL, and the blended solutions with PUL, are shown in Figure 1. The blended solutions contained 7.5% (wt/wt) of either CAS and 7.5% (wt/wt) PUL, for a total of 15% (wt/wt) CAS-PUL solution. Electrospinning of the CaCAS and NaCAS solutions (Figures 1A and 1B) produced only droplets, while the PUL solution (Figure 1C) produced some fibers with large beads, similar to previous results (Liu et al., 2016; Tomasula et al., 2016). In aqueous solution, the phosphoserine groups in CaCAS are joined by Ca bridges that formed when acid casein was converted to CaCAS during manufacture. The resultant dense protein chains likely prevented some or all entanglement. On the other hand, NaCAS form small fibers and agglomerates when dissolved in water (McMahon and Oomen, 2013) and are also unable to entangle.

**Figure 1:**
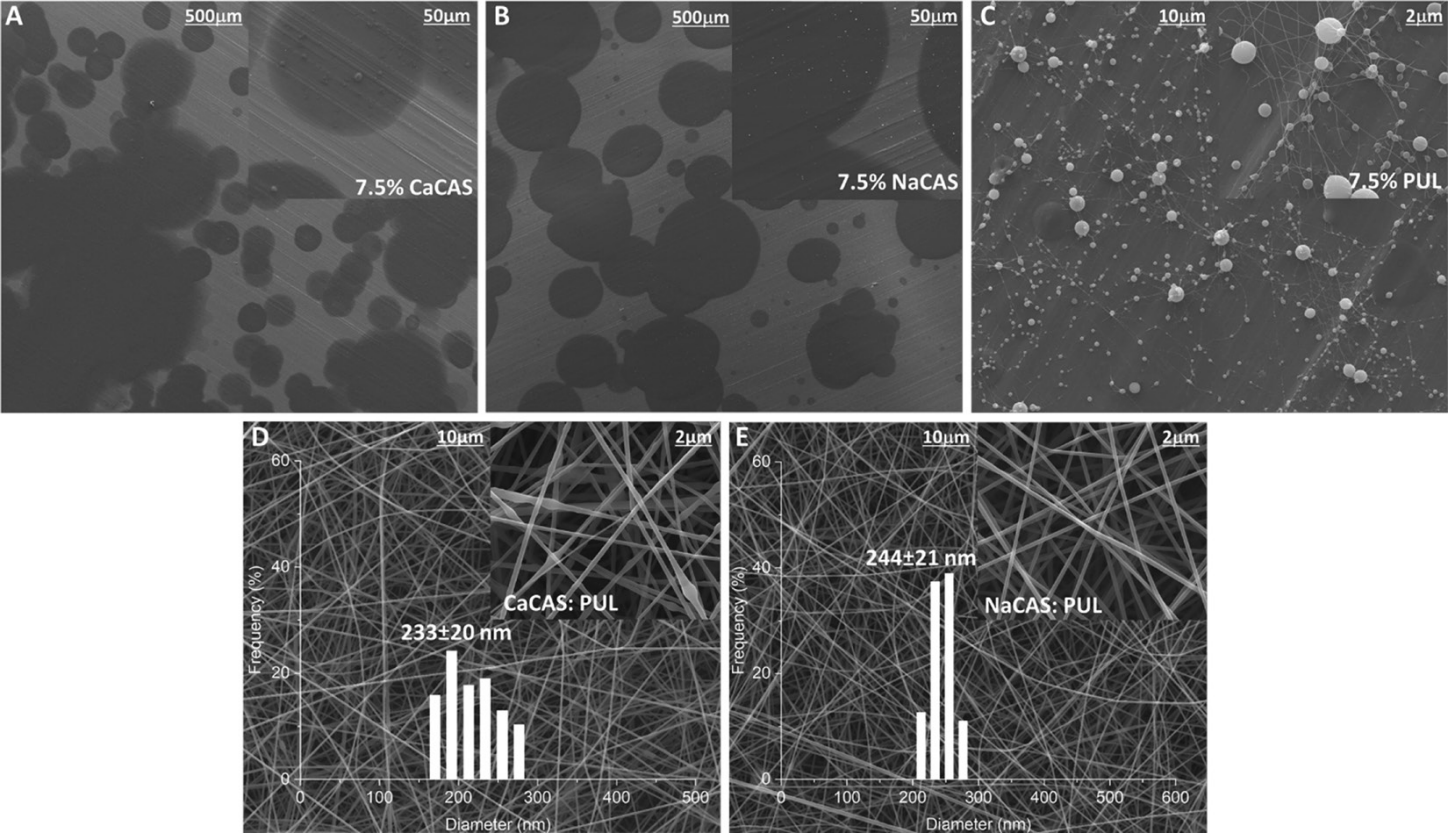
Scanning electron microscopy images of electrospun fibers in mats produced at 21°C from aqueous solutions (A) 7.5% (wt/wt) calcium caseinate (CaCAS), (B) 7.5% (wt/wt) sodium caseinate (NaCAS) at magnifications of 100× and 1,000×, (C) 7.5% (wt/wt) pullulan (PUL), (D) 7.5% (wt/wt) CaCAS-7.5% (wt/wt) PUL, 233 ± 20 nm, and (E) 7.5% (wt/wt) NaCAS-7.5% (wt/wt) PUL, 244 ± 21 nm at magnifications of 5,000× and 25,000× (*P* < 0.05).

Addition of PUL to the CAS solutions increased the shear viscosities of the CaCAS and NaCAS solutions from 0.01 and 0.02 Pa·s, to 0.17 and 0.18 Pa·s for the respective CAS-PUL solutions, enabling both chain entanglement and the subsequent increase in viscosity for fiber formation (Table 1). This confirms our previous hypotheses that addition of positive ions to PUL most likely interrupts the hydrogen bonding of its inter- and intramolecular chains, thereby freeing the chains to extend and entangle. While the magnitudes of the electrical conductivity and ZP of PUL are almost nil in comparison to those of CAS, the surface tension of PUL is about 1.4 times that of the surface tensions of the charged CAS. As in Li et al. (2017), it was also noted that the increased viscosities of the CAS-PUL solutions were accompanied by decreases in the surface tensions of these solutions compared to that of PUL alone, so that a continuous jet was able to form and deposit fibers from the charged CAS-PUL feed solutions.

**Table 1:** Properties of the calcium caseinate (CaCAS), sodium caseinate (NaCAS), and pullulan (PUL) solutions, and respective CAS-PUL blends (mean ± SD)^1^

Solution	Ratio of mixing (%)	CAS solution in the mix (%)	PUL solution in the mix (%)	TS concentration (%)	pH	Shear viscosity at 100 s^−1^ (Pa·s^−1^)	Electrical conductivity (mS·cm^−1^)	Surface tension (mN·m^−1^)	Zeta potential (mV)	Morphology/fiber diameter size (nm)	Initial load of LGG in CAS-PUL suspension, log_10_(cfu·mL^−1^)	Viability of LGG in CAS-PUL fibers, log_10_ (cfu·g^−1^)
CaCAS	1:0	7.5	0	7.5	7.1	0.01	0.91^a^	44.93 ± 0.0^a^	−12.0 ± 0.9^a^	No fiber	—	—
NaCAS	1:0	7.5	0	7.5	6.8	0.02	2.06^b^	46.95 ± 0.0^b^	−22.2 ± 1.8^b^	No fiber	—	—
PUL	0:1	0	7.5	7.5	5.3	0.05	0.03^c^	64.78 ± 0.1^c^	−2.8 ± 0.0^c^	Beads	—	—
CaCAS-PUL	1:1	7.5	7.5	15	7.1	0.17	0.69^d^	46.15 ± 0.0^d^	−16.9 ± 0.5^d^	Fibers/233 ± 20^a^	—	—
NaCAS-PUL	1:1	7.5	7.5	15	6.7	0.18	1.65^e^	47.99 ± 0.0^e^	−25.1 ± 0.5^e^	Fibers/244 ± 21^a^	—	—
CaCAS-PUL with LGG	1:1	7.5	7.5	15	6.5	0.21	0.91^f^	45.68 ± 0.0^f^	−12.6 ± 0.6^a^	Fibers/212 ± 14^a^	9.3 ± 0.7^a^	9.5 ± 1.1^a^
NaCAS-PUL with LGG	1:1	7.5	7.5	15	6.1	0.17	1.78^g^	47.37 ± 0.0^g^	−7.3 ± 0.7^f^	Fibers/286 ± 16^b^	9.0 ± 0.4^a^	9.6 ± 0.8^a^

a–g Means within a column with different superscripts differ (*P* < 0.05).

1 The *Lactobacillus rhamnosus* GG (LGG) results were obtained in triplicates. CaCAS-PUL = the blend solution from neat solutions of 15% (wt/wt) CaCAS and 15% (wt/wt) PUL with a 1:1 weight mixing. NaCAS-PUL = the blend solution from neat solutions of 15% (wt/wt) NaCAS and 15% (wt/wt) PUL with a 1:1 weight mixing.

Electrospinning of the CaCAS-PUL and NaCAS-PUL solutions produced fibers with diameters of 233 ± 20 nm and 244 ± 21 nm (*P* < 0.05), respectively (Figures 1D and 1E). While there was no difference in size between the CAS fibers, examination of Figure 1D shows strings of beads within the fibers. These beads may be related to the interactions of CaCAS and NaCAS with the OH group of PUL through hydrogen bonding as described above that were then drawn closer together during drying as water is evaporated from the fibers (Tomasula et al., 2016; Li et al., 2017).

Next, inoculation of the CaCAS-PUL blend viscous solution resulted in a cell suspension that contained 9.3 log_10_ cfu·mL^−1^ of LGG, whereas the NaCAS-PUL solution contained 9.0 log_10_ cfu·mL^−1^. The shear viscosities of the solutions at 100 s^−1^ showed little change upon addition (*P* < 0.05) of LGG (Table 1), but decreases of 0.4 and 0.7 pH units were noted in the CaCAS-PUL-LGG and NaCAS-PUL-LGG solutions. In addition, the electrical conductivities and ZP increased in value compared with the CAS-PUL values, indicating a weakening of the interactions of CaCAS and NaCAS with PUL due to the introduction of the surface negative LGG.

Bioactive LGG cells with lengths in the range from 1.1 to 5.1 µm were spotted both within fibers, as double cell strains, and in the space around the fibers, as shown in Figure 2. The CaCAS-PUL-LGG fibers had a mean diameter of 212 ± 14 nm and were not different than the fibers without LGG. The NaCAS-PUL-LGG fibers had a mean diameter of 286 ± 16 nm and were different than the other fibers with or without LGG (Table 1).

**Figure 2:**
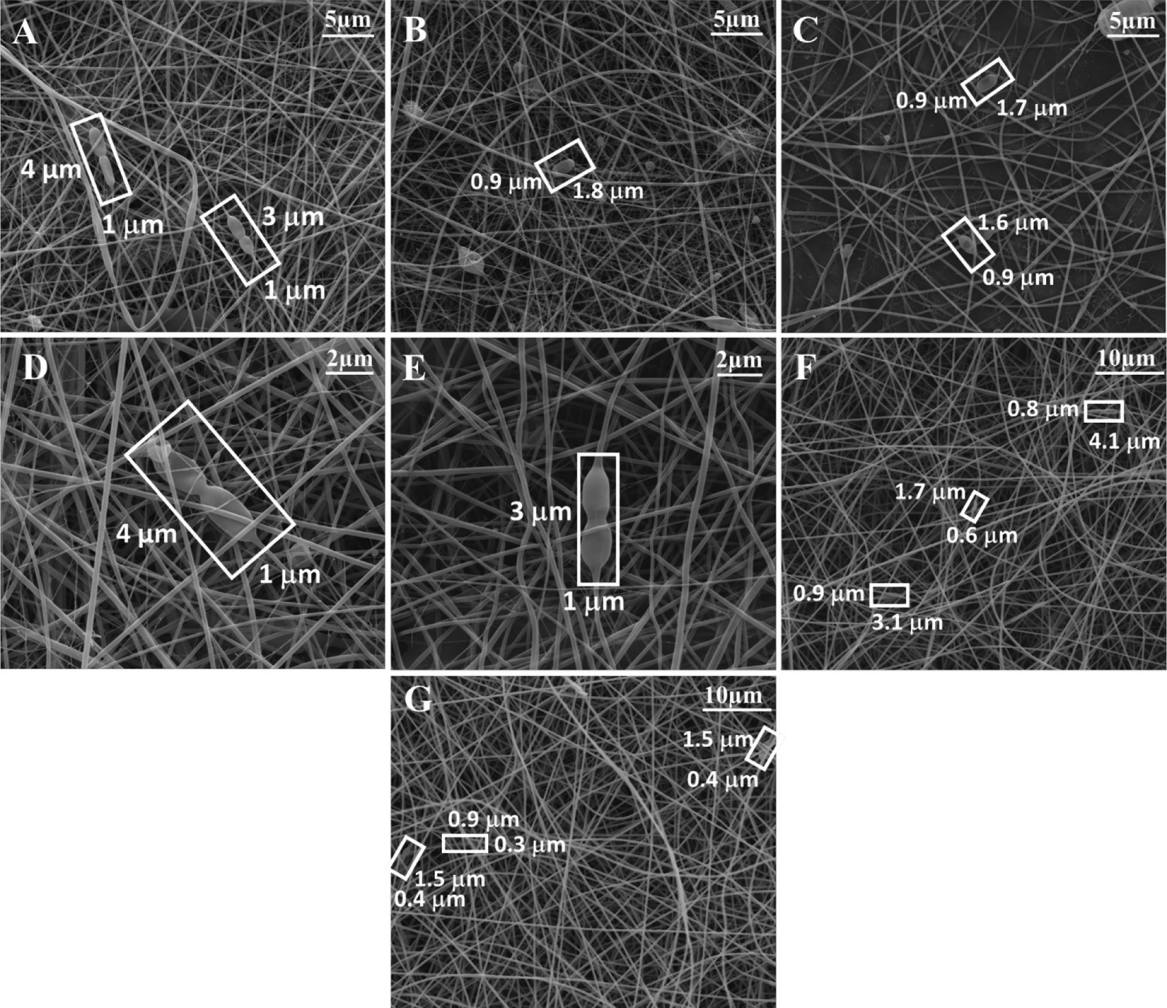
Scanning electron microscopy images of electrospun *Lactobacillus rhamnosus* GG (LGG) incorporated in fibrous mats from CaCAS-PUL-LGG [7.5% (wt/wt)-7.5% (wt/wt)-log_10_ 9.3 cfu/mL] and NaCAS-PUL-LGG [7.5% (wt/wt)-7.5% (wt/wt)-log_10_ 9.0 cfu/mL] fibrous mats. Fiber diameters are (A) 200 ± 31^a^, (B) 209 ± 32^a^, and (C) 228 ± 30^a^ nm for CaCAS-PUL-LGG fibers with porosities of 46, 47, and 47%, respectively, magnified 10,000×, (D) 127 ± 30^b^, and (E) 125 ± 25^b^ nm for CaCAS-PUL-LGG fibers with porosities of 42 and 48%, respectively, magnified 25,000×, and (F) 286 ± 18^c^ and (G) 298 ± 14^c^ nm for NaCAS-PUL-LGG fibers with porosities of 52 and 48%, respectively, magnified 5,000×. ^a–c^Means with different superscripts differ (*P* < 0.05). CaCAS = calcium caseinate; NaCAS = sodium caseinate; PUL = pullulan.

The number of viable LGG recovered from the nanofibrous mats after electrospinning was 9.5 and 9.6 log_10_ cfu·g^−1^, respectively (Table 1). The LGG cells with diameters larger than the fiber diameters were completely coated and oriented along with the nanofibers with diameters in the parts of coated probiotics of 777 ± 258 nm (Figure 2). Also, some interactions occur between the cells and proteins since it is reported that the LGG cells have a high affinity towards milk proteins such as caseins via hydrophobic interactions or van der Waals bonds (Burgain et al., 2013b). Additionally, LGG has also been shown to adhere to whey proteins, specifically β-LG, via its cell surface pili (Burgain et al., 2013b; Guerin et al., 2016) and these interactions have resulted in an increased encapsulation efficiency in dairy matrices (Burgain et al., 2014) and improved survival of the probiotic during transit within the gastrointestinal tract (Burgain et al., 2013a). In LGG mutants without surface pili, interactions were observed between whey proteins and other cell surface proteins and exopolysaccharides, but no interactions with caseins were identified (Guerin et al., 2018). As only micellar caseins and whey proteins were used in previous work, more work is needed to determine the specific interactions for NaCAS and CaCAS, which would also involve consideration of the electrostatic interactions with the cell surface components required for the localization of LGG along or within the nanofibers.

In conclusion, this is the first example of encapsulation of LGG into the electrospun fibers produced from aqueous milk protein CAS blended with PUL solutions. These findings demonstrate the potential for electrospun fibers and fibrous mats to employ this type of application, even though more extensive studies are required to determine the true potential of using the fibrous mat as a vehicle for the delivery of LGG on real food use.
